# Pb nanospheres in ancient zircon yield model ages for zircon formation and Pb mobilization

**DOI:** 10.1038/s41598-019-49882-8

**Published:** 2019-09-23

**Authors:** Ian C. Lyon, Monika A. Kusiak, Richard Wirth, Martin J. Whitehouse, Daniel J. Dunkley, Simon A. Wilde, Dirk Schaumlöffel, Julien Malherbe, Katie L. Moore

**Affiliations:** 1 Department of Earth and Environmental Sciences, University of Manchester, Manchester, UK; 2 Photon Science Institute, University of Manchester, Manchester, UK; 3 Institute of Geological Sciences, Polish Academy of Sciences, PL-00818 Warsaw, Poland; 4 GeoForschungsZentrum, Section 3.6 Chemistry and Physics of Earth Materials, D-14473 Potsdam, Germany; 5 GeoForschungsZentrum, Section 3.5 Interface Geochemistry, D-14473 Potsdam, Germany; 6 Swedish Museum of Natural History, SE-104 05 Stockholm, Sweden; 7 Faculty of Earth Sciences, University of Silesia in Katowice, PL-41205 Sosnowiec, Poland; 8 School of Earth and Planetary Sciences, Curtin University, PO BOX U1987, WA 6845 Perth, Australia; 9 CNRS / Université de Pau et des Pays de l’Adour, E2S UPPA, IPREM, UMR 5254, 64000 Pau, France; 10 Department of Materials, University of Manchester, Manchester, UK

**Keywords:** Geochemistry, Precambrian geology

## Abstract

Nanospheres of lead (Pb) have recently been identified in zircon (ZrSiO_4_) with the potential to compromise the veracity of U-Pb age determinations. The key assumption that the determined age is robust against the effects of Pb mobility, as long as Pb is not lost from the zircon during subsequent geological events, is now in question. To determine the effect of nanosphere formation on age determination, and whether analysis of nanospheres can yield additional information about the timing of both zircon growth and nanosphere formation, zircons from the Napier Complex in Enderby Land, East Antarctica, were investigated by high-spatial resolution NanoSIMS (Secondary Ion Mass Spectrometry) mapping. Conventional SIMS analyses with >µm resolution potentially mixes Pb from multiple nanospheres with the zircon host, yielding variable average values and therefore unreliable ages. NanoSIMS analyses were obtained of ^207^Pb/^206^Pb in nanospheres a few nanometres in diameter that were resolved from ^207^Pb/^206^Pb measurements in the zircon host. We demonstrate that analysis for ^207^Pb/^206^Pb in multiple individual Pb nanospheres, along with separate analysis of ^207^Pb/^206^Pb in the zircon host, can not only accurately yield the age of zircon crystallization, but also the time of nanosphere formation resulting from Pb mobilization during metamorphism. Model ages for both events can be derived that are correlated due to the limited range of possible solutions that can be satisfied by the measured ^207^Pb/^206^Pb ratios of nanospheres and zircon host. For the Napier Complex zircons, this yields a model age of *ca* 3110 Ma for zircon formation and a late Archean model age of 2610 Ma for the metamorphism that produced the nanospheres. The Nanosphere Model Age (NMA) method constrains both the crystallization age and age of the metamorphism to ~±135 Ma, a significant improvement on errors derived from counting statistics.

## Introduction

Geochronology is crucial to the understanding of geological processes, and SIMS (Secondary Ion Mass Spectrometry) U-Pb geochronology of zircon (ZrSiO_4_) has become the most widely used method for dating rocks that have undergone complex or multiple thermal events. Zircon contains trace amounts of three Pb-producing radionuclides: ^238^U → ^206^Pb, ^235^U → ^207^Pb and ^232^Th → ^208^Pb. Because ^238^U and ^235^U have half-lives of 4468 Ma and 704 Ma, respectively, ^238^U/^206^Pb and ^235^U/^207^Pb give independent age estimates. Concordance between these ages (and with ^207^Pb/^206^Pb) provides evidence that the U-Pb decay system has not been disturbed. However, U-Pb systematics of many zircons are disturbed, due to Pb mobility during geological processes such as metamorphism, fluid infiltration and weathering^1–7^.

The mechanism for such disturbance is initiated by radiation damage of zircon resulting from radioactive decay of U and Th, creating locally amorphous domains that contain the stable daughter isotopes of Pb^8,9^. With sufficient time and actinide concentration, the zircon becomes metamict, where amorphous domains overlap to form a continuous Pb-bearing network of damaged zircon between islands of crystalline zircon. A transmission electron microscope (TEM) study of zircons from the granulite-facies Napier Complex in Enderby Land, east Antarctica^10^, showed that Pb mobilization occurred during metamorphism, forming crystalline native Pb nanospheres typically <10 nm in diameter and 100s of nanometers apart. The authors interpreted this phenomenon as resulting from Ostwald ripening of radiogenic Pb accumulations during thermal annealing of metamict zircon. The formation of metal Pb nanospheres therefore effectively isolated them from further ingrowth of radiogenic Pb and from subsequent Pb loss. After metamorphism, continued U and Th decay resulted in the renewed accumulation of radiogenic Pb in the zircon between the Pb nanospheres. The migration of radiogenic Pb in zircon during metamorphism has been established by several techniques, including SIMS^6,11–15^, TEM^10,16,17^ and atom-probe tomography (APT)^18–21^.

Conventional SIMS analyses at 10 μm lateral resolution sample variable mixtures of Pb from nanospheres and from the zircon host, potentially giving unreliable ages. For example, Kusiak *et al*.^11^ demonstrated that ^207^Pb/^206^Pb ratio measurements of ~10 µm spots of concentrated radiogenic Pb yielded apparent ages ranging between 4.15 and 2.76 Ga from measurement sites only tens of micrometers apart in a zircon whose ^207^Pb/^206^Pb crystallization age was determined as 3.38 Ga by SIMS. This analysis scale is however still ~10^3^ larger than typical nanospheres. In an alternative approach, Valley *et al*.^18,19^ and Peterman *et al*.^20^ used APT to analyze ^207^Pb/^206^Pb from a small number of Pb clusters (~10^3^ Pb atoms) trapped in volumes of zircon ~10^3^ nm^3^ and were able to show that ^207^Pb/^206^Pb ages could be deconvoluted using a Tera-Wasserburg plot to yield the zircon crystallization age and the Pb mobilization age. These authors concluded that the formation of Pb clusters had no influence on the U-Pb age. However, the situation is different in zircon from the Napier Complex in Antarctica, where areas containing metal Pb nanospheres, when analyzed by SIMS, gave variable ages without geological meaning. To solve this problem, we utilized NanoSIMS and hereby demonstrate that high spatial resolution mapping of large areas of zircon >100 µm^2^, with sufficient spatial resolution to analyze many individual nanospheres within that area, can directly yield both the zircon formation age and the timing of metamorphism that caused the Pb mobilization.

### Theory and constraints on Nanosphere Model Ages (T_NMA_)

Measurement of ^207^Pb/^206^Pb allows a determination of age that is not subject to the uncertainties associated with U-Pb isotope calibrations if it can be assessed that there was no initial Pb in the zircon^22^. Nevertheless, the relationship between ^207^Pb/^206^Pb and its calculated age depends on maintaining the association between daughter and parent isotopes to the present day. If radiogenic Pb was dissociated from its uranium parent in the past, then its ^207^Pb/^206^Pb composition will not correspond to a ^207^Pb/^206^Pb ‘age’ as conventionally calculated.

The ^207^Pb/^206^Pb composition in this case may be derived if we assume that the zircon is formed at time T_1_ and with no inherited Pb. Uranium and Th decay in the zircon produce domains of radiation damage in the zircon lattice. At time T_2_, an event occurred which mobilized the Pb accumulated from U and Th decay within the zircon between T_1_ and T_2_. Such Pb mobilization most likely occurred during high-temperature metamorphism of the rocks in which the zircon resides, but can occur through other processes too, such as fluid-assisted coupled dissolution-reprecipitation, although the latter is more likely to remove radiogenic Pb and/or contaminate the zircon with Pb from outside the grains^23^. Mobilization causes Pb to aggregate into nanospheres as the Pb-incompatible crystal lattice of the zircon host is repaired. If there are no further Pb mobilization events and with no U or Th in the nanospheres, the ^207^Pb/^206^Pb composition of the nanospheres remains constant after T_2_. The Pb isotopic composition of the nanosphere formed at time T_2_ is therefore ‘frozen’ and the nanosphere represents an isotopic ‘fossil’ of its time of formation. Subsequent U and Th decay in the zircon host around the nanospheres between T_2_ and today produces further radiogenic Pb, and if the U and Th distribution is homogeneous then nanoscale observation reveals Pb nanospheres surrounded by radiation damaged zircon with a homogeneous distribution of radiogenic Pb.

The system may be understood using the Holmes-Houterman model^22^ in which the zircon formed at time T_1_ and the Pb was mobilized at time T_2_, with the ^207^Pb/^206^Pb ratio in the zircon at the time of Pb mobilization given by:

1 $$(\frac{207Pb}{206Pb})\ast =\frac{1}{137.82}[\frac{{e}^{\lambda 235T1}-{e}^{\lambda 235T2}}{{e}^{\lambda 238T1}-{e}^{\lambda 238T2}}]$$

which will be the ^207^Pb/^206^Pb ratio retained within the nanospheres to the present day. The ratio 1/137.82 relates to the present day ^235^U/^238^U ratio, and λ_235_ and λ_238_ represent the decay constants for ^235^U and ^238^U, respectively. Subsequent U decay within the zircon host will produce a ^207^Pb/^206^Pb ratio representing radiogenic Pb accumulation until the present day^22^.

2 $$(\frac{207Pb}{206Pb})\ast =\frac{1}{137.82}[\frac{{e}^{\lambda 235T2}-1}{{e}^{\lambda 238T2}-1}]$$

**Figure 1:**
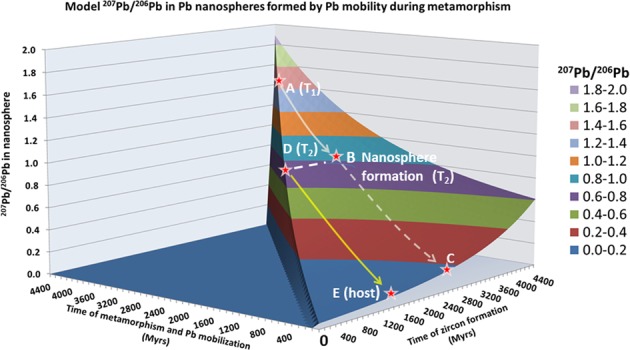
Surface of ^207^Pb/^206^Pb compositions defined by Eq. 1 for all possible times T_1_ (zircon formation) and T_2_ (time of Pb mobilization). For a zircon formed at time T_1_ (point A), the ^207^Pb/^206^Pb composition in the zircon will evolve along the track towards point C and this will be the isotopic composition of the Pb averaged over the whole zircon if measured today. If at time T_2_ there is an event that mobilizes the Pb into nanospheres by clearing Pb out of the metamict host and isolating the nanospheres from further U decay, then the ^207^Pb/^206^Pb composition of the nanospheres will remain ‘frozen’ at point B. The host zircon surrounding the nanospheres, emptied of Pb at T_2_ will gain Pb by subsequent U (and Th) decay from point D and its isotopic composition will evolve to point E at the present day.

In this general model, the isotopic composition of radiogenic Pb that has accumulated from time T_1_ until T_2_ when the nanospheres form, and the evolution of the composition of radiogenic Pb in the zircon host due to subsequent actinide decay from T_2_ until the present day, is portrayed graphically in Fig. 1. A consequence of this model is that the ^207^Pb/^206^Pb ratio frozen into the nanospheres will be in the range of 2.007 > ^207^Pb/^206^Pb > 0.0464 for 4.54 Ga > T_1_ > T_2_ > 0, whereas the Pb within the host zircon formed after the Pb mobilization event will range from 0.618 > ^207^Pb/^206^Pb > 0.0464. The time T_2_ can be determined from the Pb isotopic composition of zircon in between nanospheres and if T_2_ may be determined, then T_1_ may be calculated from the Pb isotopic composition of the nanospheres.

In general however, with error limits on measured ^207^Pb/^206^Pb values for the nanospheres and zircon host, there is a limited, correlated range of possible values for T_1_ and T_2_ which can satisfy Eqs. 1 and 2. We term the model age solutions of this method the Nanosphere Model Age (T_NMA_).

There is an additional constraint that may be placed on T_1_ and T_2_: the quantity of Pb available for formation of the nanospheres is sensitive to the time difference (T_1_ − T_2_) and hence the proportion of nanosphere-hosted Pb relative to Pb in the zircon host provides an additional constraint on possible model solutions to T_1_ and T_2_ from the experimental data. Although T_2_ is primarily constrained by the ^207^Pb/^206^Pb composition in the zircon host, the uncertainty on this measurement yields a range of possible T_1_ values from Eq. (1). However, not all of these possible T_1_ values predict nanosphere ^207^Pb/^206^Pb values which fall within the range of experimental measurements. In other words, the constraint placed by (T_1_ − T_2_) on possible values for the ^207^Pb/^206^Pb in the nanospheres places additional constraints on the model age for T_2_. Model ages for the crystallization age and metamorphism are correlated and precision is better on the determined model T_1_ and T_2_ ages are better than the individual measurements of ^207^Pb/^206^Pb of nanospheres and zircon outside of nanospheres might imply. This is illustrated in Fig. 1 of the supplementary information.

The key assumptions in this method are that (1) all of the radiogenic Pb within the zircon at T_2_ is moved into nanospheres during the Pb mobilization event, so that no Pb remains outside of nanospheres dispersed in the zircon at T_2_; and (2) that there is no loss of Pb from the zircon to the host rock during the Pb mobilization event. If assumption (1) is invalid, the Pb isotope composition of nanospheres will remain as determined by Eq. (1) but the ^207^Pb/^206^Pb of the zircon host will be higher than predicted by T_2_ alone. If assumption (2) is invalid then the ^207^Pb/^206^Pb ratios of nanospheres will remain as predicted by Eq. (1), and the model ages T_1_ and T_2_ would be unaffected, but the amount of Pb in nanospheres compared to the amount in the zircon host measured today would be affected.

A significant consequence of the model is that for all values of T_1_ and T_2_ during Earth history (where T_1_ > T_2_ > 0) the ^207^Pb/^206^Pb ratio of the nanospheres and of radiogenic Pb in the host zircon, as measured today, would be different, leading to significant ^207^Pb/^206^Pb heterogeneity on a nanometer scale within the zircon wherever Pb has been redistributed into clusters or nanospheres at some time in the past^6^.

## Materials and Methods

Zircon grains for the present study (see sample preparation details in references^9,10^) came from a granulite-facies orthogneiss from Gage Ridge (rock sample 975, grains 07 and 66) in the Napier Complex of East Antarctica. At least two high-grade metamorphic events have been recognized in the area. Low-P and high-T metamorphism occurred at *ca* 2.8 Ga, followed by an ultra-high-temperature (UHT) event at *ca* 2.55 Ga, with temperature estimates as high as 1100 °C^24–27^ for the latter.

### TEM

Four foils across grains 07 and 66 (2 foils from each) were prepared for TEM study to verify the Pb nanosphere distribution from rim to core. The site-specific focused-ion-beam (FIB) technique, which allows cutting an electron-transparent foil from pre-selected areas of interest, was applied^28,29^. The TEM foils were 15–20 μm wide, 10–15 μm deep and 150 nm thick. A glass fibre attached to a micro-manipulator was used to lift out foils from the excavation sites. Details of the technique are given in Wirth^30^. Analytical and energy-filtered high-resolution transmission electron microscopy (ATEM, HRTEM) using a FEI Tecnai^TM^ G2 F20 X-Twin at GeoForschungs Zentrum (GFZ) in Potsdam, operated at 200 kV with a field emission gun (FEG) electron source, was used for the present study. The TEM is equipped with a post-column Gatan imaging filter (GIF Tridiem). The HRTEM images presented were energy-filtered using a 10 eV window on the zero loss peak. ATEM was performed with an EDAX X-ray analyzer equipped with an ultra-thin window. The X-ray intensities were measured in scanning transmission mode (STEM) where the electron beam was serially scanned over a pre-selected area, minimizing mass loss during data acquisition.

### NanoSIMS mapping

Previous SIMS ^207^Pb/^206^Pb dating of zircons utilized large primary ion beams (on the nA scale) and consequently spatial resolutions of ~10–20 µm were required in order to generate sufficiently large Pb^+^ secondary ion signals for analysis. This is a consequence of the Pb abundance averaged over the zircon being at the ppm level. However, as the Pb nanospheres are Pb metal, if the primary SIMS ion beam can be made of a similar dimension to the nanosphere, then measurable Pb^+^ secondary ion signals may be generated^31^. This is achieved by the NanoSIMS where the primary ion beam can be focused to a spatial resolution of 50–100 nm.

The University of Manchester NanoSIMS was here equipped with a duoplasmatron source for O^−^ (minimum primary ion spot size 100 nm) and the University of Pau NanoSIMS was equipped with a RF-Plama O^-^ ion source for high brightness and spatial resolution of 50 nm. A primary ion beam of O^−^ was used to sputter positive secondary ions in imaging mode over areas between 100 × 100 μm^2^ and 1 × 1 μm^2^.

At ~2 × 10^2−1^ × 10^6^ Pb atoms per nanosphere and a detection efficiency of ~5 × 10^−3^ this leads to a total detected number of Pb^+^ ions of between ~1 to 1 × 10^3^ per nanosphere, so the precision on measured ^207^Pb/^206^Pb from individual nanospheres will be limited by counting statistics. The detection efficiency was calibrated from the total Pb^+^ secondary ion current compared to the number of Pb atoms sputtered from a Pb metal standard.

The Manchester and Pau NanoSIMS are each equipped with a multicollector for the simultaneous acquisition of secondary ion beams. The highest mass at which single mass unit spacing may be achieved is 56 which meant that ^204^Pb^+^ could be measured in multicollection mode with ^208^Pb^+^, but ^206^Pb^+^ and ^207^Pb^+^ could only be measured by magnetic peak switching on a single detector. The measurement cycle in imaging mode therefore acquired ^206^Pb^+^, then ^207^Pb^+^, and then the simultaneous acquisition of ^27^Al^+^, ^40^Ca^+^, ^48^Ti^+^, ^89^Y^+^, ^190^HfO^+^, ^204^Pb^+^ and ^208^Pb^+^. This procedure was changed for some data acquisitions where detector 7 was used to acquire ^206^Pb in multicollection with the other species and used magnetic peak switching to acquire ^207^Pb. This had the advantage of more time spent on acquiring ^207^Pb and ^206^Pb but the disadvantage of not acquiring measurements of ^204^Pb and ^208^Pb. Repetition of this cycle until the nanospheres were completely sputtered away allowed the determination of ^207^Pb/^206^Pb for individual nanospheres by defining regions of interest (ROIs) around the Pb nanospheres in the isotope images and extracting counts for the different measured species. Automated algorithms for particle detection based on threshold intensity within the acquired images were used in L’Image (NanoSIMS image processing software, L Nittler) to define areas of ^207^Pb and ^206^Pb clustering. After defining the ROIs, these areas of Pb concentration were subtracted from the whole image to define a host zircon ROI which contained ^207^Pb and ^206^Pb without detectable Pb concentrations.

The data acquisition and processing procedures were tested by imaging 100 µm^2^ areas of a NIST 981 Pb metal standard and dividing the area into 10^4^ 100 × 100 nm^2^ square ROIs to obtain ^204^Pb/^206^Pb, ^207^Pb/^206^Pb and ^208^Pb/^206^Pb for each ROI. Reproducibility of Pb isotope ratios are within 11‰ for ^204^Pb/^206^Pb, 8‰ for ^207^Pb/^206^Pb and 4‰ for ^208^Pb/^206^Pb of the certified NIST 981 ratios^22^ and within the counting statistic errors at the 2σ level. This procedure demonstrated that the precision on acquired ^204^Pb/^206^Pb, ^207^Pb/^206^Pb and ^208^Pb/^206^Pb measurements on zircon samples was limited by counting statistics alone and that there were no systematic errors at this level of precision which affected the accuracy of the measured ratios.

An assessment was made of the initial Pb content of the zircon by measuring the abundance of ^204^Pb, and of ^208^Pb formed through the decay of ^232^Th and these data are presented in Table 1 in the supplementary information.

### NanoSIMS depth profile

A 3-dimensional depth profile of a volume 1 μm × 0.7 μm × 0.3 μm was acquired by rastering a 50 nm diameter beam across an area of 1 μm × 1 μm and sputtering into the sample. The result is shown in Fig. 4. The image was acquired with 64 × 64 pixels = 15 nm pixel^−1^. Drift correction and data analysis was carried out using L’Image software (L. Nittler) and used to produce images of 87 individual sample planes with 3 pixels smoothing in each detected species to match the primary ion beam spot size. A composite image of the stack of 87 images formed by merging the images of ^27^Al, ^48^Ti and ^206^Pb (Fiji image analysis software, https://fiji.sc/) was used to generate the 3-dimensional image. The depth axis has been stretched by a factor of 3 to show the relative positions of these species within the volume.

## Results

**Figure 2:**
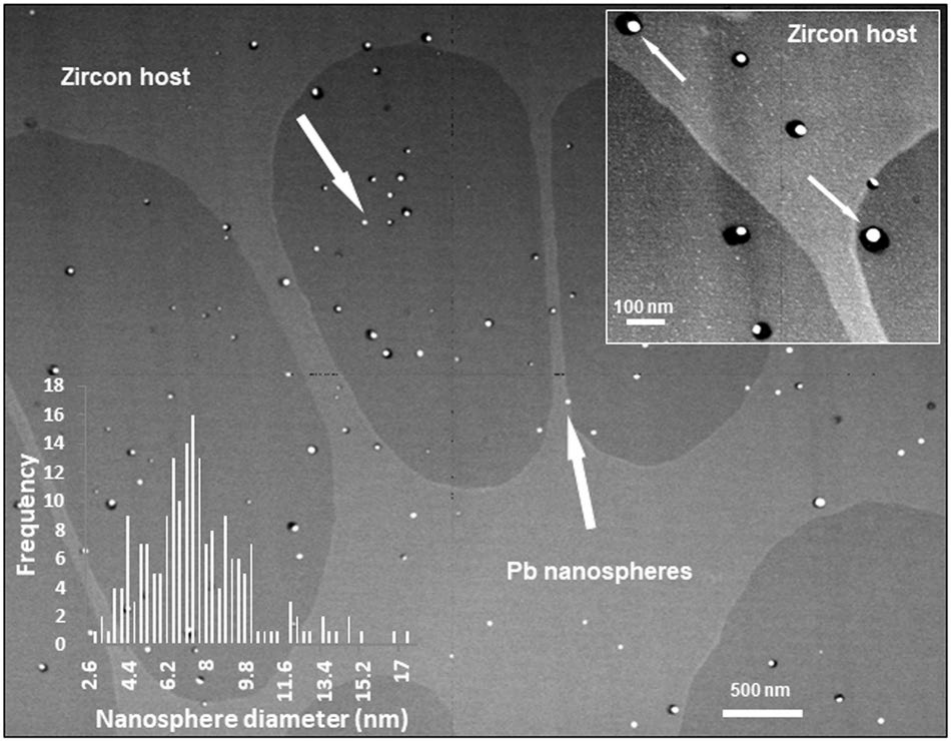
High-angle annular dark-field (HAADF) TEM image showing Pb nanospheres (white) in zircon host (grey background; lighter grey background due to increased contrast from underlying Cu support grid) in sample 975 (foil #3184). Arrows point to some of the Pb nanospheres. Black ‘mantle’ around certain white Pb nanospheres is a Si-rich phase. The inset in the top right corner shows the Pb nanospheres at a larger scale; the inset in the bottom left corner shows the size distribution of the nanospheres calculated from the image.

**Figure 3:**
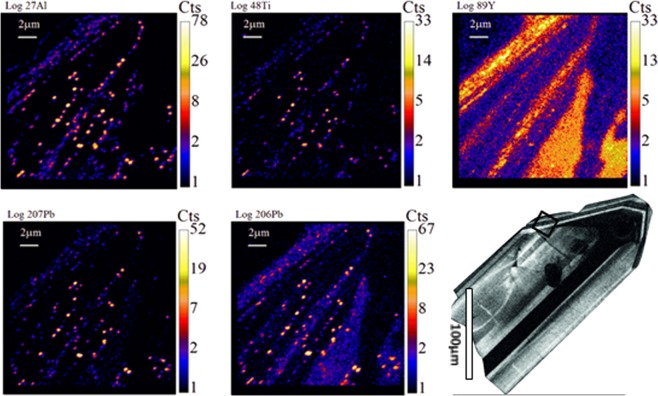
NanoSIMS imaging showing relative intensities of ^27^Al, ^48^Ti, ^89^Y, ^206^Pb, ^207^Pb and ^208^Pb in zircon grain 975, grain identifier n3850–66. Field of view is 20 µm × 20 µm. Colour bars give maximum counts per pixel for each species and are presented as log (counts) to bring out the low intensity structure, particularly of zircon host areas which have higher ^206^Pb abundance compared with nanospheres which have a higher ^207^Pb/^206^Pb ratios. On the bottom right side, the CL image of the zircon grain is presented with the black square indicating the area where the ion image maps were taken.

**Figure 4:**
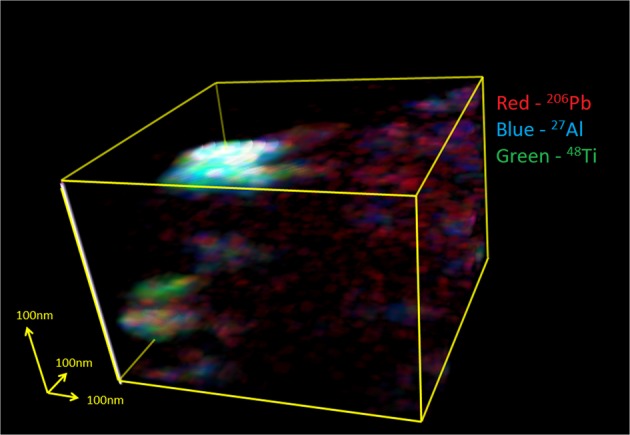
Reconstruction of a 3D volume of sample 975, grain 66 acquired by depth profiling into a 1 μm x 1 μm area on the surface of the zircon to a depth of 0.3 μm and recording ion signals as a function of depth. Drift of the image during the acquisition limited the volume over which images were acquired for the whole depth profile to 1 μm × 0.7 μm × 0.3 μm; red ^206^Pb, blue ^27^Al, green ^48^Ti. Where these overlap strongly they sum to white. Depth scale was estimated from SEM images of the crater post-analysis and in this figure has been stretched by x3 compared to the other two axes to show more clearly the relationships between components within the volume. Details of imaging processing to produce this figure are given in the Methods section.

Our TEM data confirm that Pb nanospheres in zircon are present regardless of zircon growth zonation, cracks or inclusions (Fig. 2). Lead nanospheres are heterogeneously distributed and of various sizes. They occur either as individual inclusions directly in the zircon host or together with a Si-rich phase (visible as a black mantle around the white Pb nanospheres in Fig. 2), locally together with an Al-Ti phase (not visible on Fig. 2). Isotope maps acquired by NanoSIMS mapping show that not only is Pb heterogeneously distributed (sharply defined nanospheres of ^206^Pb and ^207^Pb), but there is an association of ^27^Al and ^48^Ti with some of Pb nanospheres (Fig. 3). The Ca^+^ images (Fig. 2 in supplementary information; SI) showed a high background level, heterogeneous distribution that was interpreted as contamination of the sample surface by Ca-bearing contaminants and so is not further considered here. The distribution of ^89^Y reveals zonation characteristic of magmatic zircon growth. Depth profiles were obtained in order to characterize both the Pb nanospheres at the exposed surface of the grain and at depth. A 3D reconstruction of a 1 × 0.7 × 0.3 µm^3^ volume obtained by depth profiling a 1 µm^2^ area confirms the heterogeneous distribution of the Pb nanospheres both horizontally and vertically and shows that they are not only at the surface of the grain but at depth (Fig. 4). An important result from the depth profiling is that clustering of Pb does not always co-cluster with Ti and/or Al.

Compiled measurements of ^207^Pb/^206^Pb values for resolved Pb nanospheres and for Pb in the host zircon are presented in Table 1. The ^204^Pb/^206^Pb values measured in the nanospheres and in the zircon host (Table 1, SI) were typically zero, resulting from no detected counts of ^204^Pb within those ROIs. Detector noise counts are typically ~0.5 s^−1^ but in imaging mode are spread over 256 × 256 pixels, the probability of a noise count appearing within the ROI defining a nanosphere (typically 6–10 pixels) is ~10^−2^ over a typical data acquisition period. This allows us to place an upper limit of ^204^Pb/^206^Pb < 0.003 measured within nanospheres and assuming a ^204^Pb/^206^Pb = 0.079 in the Earth at 3 Ga, an upper limit on the amount of inherited Pb as <4%. This places an upper limit on the possible systematic error on ^207^Pb/^206^Pb in nanospheres as +0.02 due to possible inherited Pb which is smaller than the confidence limits on ^207^Pb/^206^Pb given by counting statistic precision. This demonstrates that inherited Pb from before the zircon formed in these cases were negligible, having no significant effect upon the Nanosphere Model Ages. Measurements of ^208^Pb/^206^Pb for individual zircons are also given in Table 1, SI. These are consistent with the measured Th/U = 0.05 for grain 66 and 0.06 for grain 07^12^. Thus, the assumptions of Eqs (1) and (2) are valid.

**Table 1:** Compiled ^207^Pb/^206^Pb measurements acquired from nanospheres and from zircon host in between the nanospheres.

Sample	^207^Pb/^206^Pb nanospheres	^207^Pb/^206^Pb zircon host	Model age zircon formation T_1_	Model age metamorphism T_2_	Age metamorphism determined from zircon host only
975 grain 07	0.495 ± 0.112 s.e.m. ± 0.016	0.195 ± 0.010 St. err ± 0.0044	3045 (±95) Ma	2640 ± 100 Ma	2550 ± 158 Ma
975 grain 66	0.528 ± 0.08 s.e.m. ± 0.012	0.170 ± 0.026 St. err ± 0.008	3225(±75) Ma	2560 ± 90 Ma	2770 ± 150 Ma

Errors are 2σ and derived from the standard error of 40 nanosphere analyses and 5 zircon host analyses from grain 07 and 50 nanosphere analyses and 10 zircon host analyses from grain 66. Data from individual nanospheres are shown in the supplementary information. Range in nanosphere model ages are given in brackets.

A weighted average of nanosphere ^207^Pb/^206^Pb yielded values of 0.495 ± 0.024 and 0.528 ± 0.030 (2σ error in the mean) and zircon host values of 0.170 ± 0.040 and 0.195 ± 0.052 (2σ error in the mean), along with an abundance ratio Pb(nanospheres)/Pb(host) = 0.45 ± 0.10 and 0.35 ± 0.10 for grains 66 and 07, respectively.

Model ages from these values are given in Table 1.

## Discussion

There are a number of possible systematic errors that may affect the measured ^207^Pb/^206^Pb values.

The NanoSIMS primary ion spot-size is 50–100 nm in diameter and so much larger than typical nanosphere sizes. Thus some Pb from the zircon host around the nanosphere will be analyzed along with the nanosphere, although nanospheres are spatially resolved from each other since the average separation between nanospheres is >300 nm. Some Pb^+^ ions will therefore be generated from the zircon host surrounding the nanosphere, but measured as if from the nanosphere, thereby altering the measured ^207^Pb/^206^Pb ratio and the relative Pb abundances of the nanospheres. Measurements of the intensity of the Pb^+^ signal from zircon host between nanospheres leads to an estimate that when the primary ion beam is centered on a nanosphere, the measured Pb^+^ signal from the surrounding zircon host within the primary ion beam spot will typically have a contribution of between 1–4% Pb^+^ of the nanosphere Pb^+^ signal. Potentially this systematic error is more significant for smaller nanospheres. To address whether this effect could significantly alter the measured ^207^Pb/^206^Pb ratios of the nanospheres, the measured ^207^Pb^+^ and ^206^Pb^+^ counts from the zircon host with the same primary ion beam spot size as on the nanosphere was subtracted from the ^207^Pb^+^ and ^206^Pb^+^ counts recorded from each nanosphere. Although this is an approximate estimate of the correction required, as the nanosphere will occupy some of the area of the primary ion spot, nevertheless most of the area sputtered by the beam when measuring a nanosphere will be on host zircon. The Pb^+^ signal from zircon host nm^−2^ is however considerably lower than from nanospheres so the estimated correction to the mean ^207^Pb/^206^Pb of all of the nanospheres shown in the supplementary information is ~1.2%. When the range of possible NMA is obtained from this corrected ^207^Pb/^206^Pb value, it raises the mean age of T_1_ by ~10 Ma. Although this will be a systematic error in the measured ^207^Pb/^206^Pb ratio of a nanosphere, it is nevertheless considerably smaller than the statistical error in the measurement. As the correction is an estimate and not significant to the uncertainty in the age of the nanospheres, the ages reported remain as determined from the nanosphere ^207^Pb/^206^Pb values.

If the assumption that mobilization of Pb into nanospheres completely removes Pb from the originally metamict zircon is valid, then the ^207^Pb/^206^Pb value of the latter defines the age of the Pb mobilization event (Eq. 2). The uncertainty on this age is then defined by the uncertainty in the ^207^Pb/^206^Pb value of the zircon host. A possible systematic error in this procedure is that the smallest nanospheres may not be sufficiently large to be recognized as discrete enhancements to the ^206^Pb^+^ and ^207^Pb^+^ signals compared to statistical fluctuations in the zircon host ^206^Pb^+^ and ^207^Pb^+^ signals and so cannot be recognized as nanospheres. Any small nanospheres which are therefore counted in the zircon host ^207^Pb/^206^Pb measurement may raise the mean ^207^Pb/^206^Pb value of the zircon host.

To estimate the potential contribution of small, unrecognized nanospheres to the measurement of ^207^Pb/^206^Pb of the host zircon, the measured TEM size distribution of the nanospheres in this sample may be used (inset in Fig. 2). The distribution is approximately Gaussian and has a maximum abundance at a diameter of ~7.5 nm. We estimate that a nanosphere may not be recognized as giving an enhanced signal above the zircon host when the Pb^+^ signal from the nanosphere is of the same magnitude as from the zircon host surrounding the nanosphere but still within the primary ion beam spot.

Typical nanospheres yielded ~500 counts in total of ^206^Pb^+^ and ^207^Pb^+^. From the relative volumes of spherical nanospheres, we may therefore estimate that a nanosphere of diameter 2 nm or less will give a Pb^+^ signal that would not be larger than the statistical fluctuations in the host zircon Pb^+^ signal. Inspecting the measured nanosphere size distribution (Fig. 2, inset), it can be seen that no nanospheres were identified of this size in the TEM images so we may place an upper limit on nanospheres <2.5 nm in diameter of <0.5% of the nanosphere population. Estimates by mass balance indicate that this potential systematic error is not significant in the analyses reported here compared to the statistical error on the zircon host ^207^Pb/^206^Pb measurement. However, this potential source of systematic error must nevertheless be evaluated for each acquisition.

Given the error limits on the measured ^207^Pb/^206^Pb values of both the zircon host and nanospheres, there are a range of possible values of T_1_ and T_2_ that will correspond to the measured values of ^207^Pb/^206^Pb. However an additional constraint may be placed on the possible combinations of values of T_1_ and T_2_ since the predicted ratio of Pb atom abundance in the nanospheres relative to those formed after the metamorphic event (which remain in the zircon host) is highly sensitive to (T_1_−T_2_). Thus, measurement of the Pb abundance in the nanospheres relative to Pb abundance in the surrounding host provides an additional powerful constraint upon the likely range of ages for these events. A significant assumption implicit in applying this constraint is that there was no Pb loss from the zircon during the Pb mobilization event at T_2_. Any Pb loss during this event would not have significantly altered the ^207^Pb/^206^Pb ratio of nanospheres formed at time T_2_ but could alter the ratio of Pb in the nanospheres to Pb formed after T_2_ residing in the zircon host surrounding the nanospheres.

The question arises as to the significance of the other elements that are associated with Pb mobilization in zircon from the Napier Complex in East Antarctica, namely Al, Ti and Si^10^. Although not the focus of the present study, it is pertinent to briefly discuss their distribution with respect to the Pb nanospheres and compare with other studies where radiogenic Pb concentrations have been recorded. The overall issue of Pb mobilization in zircon has been discussed in several papers utilizing various methods^10–21^, with only TEM capable of identifying crystalline metal Pb nanospheres. However, this phenomenon is not limited to zircons from the Napier Complex as metallic Pb nanospheres were also documented in the Kerala Khondalite Belt (KKB) in India^17^. What distinguishes Antarctic zircons from those in India is the presence of Al and Ti concentrations, whereas Pb nanospheres in the KKB only occur individually. Patchy distribution of Ti may have implications for Ti-in-zircon thermometry and Hadean tectonics^12,32,33^. Clustering of Al was also observed in zircons from the Napier Complex in the APT study^21^, where areas with increased Al content corresponded to increases of Y and U in individual dislocation arrays. This is not the case in the present study, as no correlation with Y or U occurs. The presence of Al in zircon from Jack Hills in Australia was also reported and explained as due to a high-Al source^34^. Furthermore, Y accumulations were also reported in association with Pb clusters in Jack Hills zircon^18,19^, but were not identified in the present study. This raises the issue of exactly how these various elements come to be concentrated in formerly metamict zircon. Currently, diverse mechanisms have been suggested, none of which fully explain the range of features exhibited by all known examples. Hence further work is required to solve what drives this phenomenon.

Zircon crystallization ages for grains 66 and 07 have been reported previously using conventional SIMS^11^. Grain 66 gave a ^207^Pb/^206^Pb age of 3344 ± 15 Ma and a ^206^Pb/^238^U age of 3162 ± 15 Ma (discordant), and grain 07 a ^207^Pb/^206^Pb age of 3609 ± 13 Ma and a ^206^Pb/^238^U age of 3705 ± 26 Ma (reversely discordant). As the Gage Ridge sample is a granulite-facies orthogneiss, it would be expected that the zircons within it were formed *in situ* and convergent crystallization ages between grains would be anticipated, whereas the ages determined by Kusiak *et al*.^11^, are not. The zircon formation model ages determined in this work from nanospheres were grain 66, 3225 Ma and grain 07, 3045 Ma. The range of possible crystallization ages which are consistent within the experimental measurement errors are ±150 Ma and so the two grains have ages concordant with the ages determined by conventional SIMS^11^ for grain 66, but not with grain 07.

A minimum age of 2.47 Ga, and probably older than 2.55 Ga, has been reported for the ultrahigh temperature metamorphic event in the Napier Complex^35^, consistent with the ages determined for the Pb mobilization event, grain 66 of 2560 ± 100 Ma and grain 07 2640 ± 90 Ma.

## Conclusions

Regardless of the formation mechanism of Pb nanospheres, their potential to affect precise U-Pb age estimates must be taken into consideration^12^. Wherever a process such as metamorphism has resulted in Pb mobilization, there exists the capability for forming Pb clusters and/or Pb nanospheres with a frozen ^207^Pb/^206^Pb ratio and values that are significantly higher than the zircon host. Since the Pb nanospheres documented here have a mean diameter of 7.5 nm, separations of typically ~300 nm and are randomly distributed, conventional SIMS ^207^Pb/^206^Pb analysis, with an analytical spot size of 10–25 μm, would sample both Pb nanospheres and the zircon host, combining them together in unpredictable proportions. This has serious implications because recent scientific advances^36^ have enabled the SIMS analytical spot size to be reduced to <10 µm. However, the high resolution measurement of individual Pb nanospheres and their host zircon by NanoSIMS, as presented above, can for the first time resolve both the time of zircon formation and the metamorphic event during which the Pb was mobilized and Pb nanospheres were formed.

## Supplementary information

Supplementary Information

## Data Availability

The data that support this study are held in University of Manchester Data Repository RDMP118 and are available on request from the corresponding author.
