# Supplementary material for: Pb nanospheres in ancient zircon yield model ages for zircon formation and Pb mobilization
Source: Sci Rep. 2019 Sep 23;9:13702. doi: 10.1038/s41598-019-49882-8 (PMC6757063; doi:10.1038/s41598-019-49882-8)
Supplement: Supplementary file 1 — Supplementary Information [file 41598_2019_49882_MOESM1_ESM.docx]

**Supplementary Information**

**Pb nanospheres in ancient zircon yield model ages for zircon formation and Pb mobilization.**

Ian C. Lyon^*1,2^, Monika A. Kusiak^3,4^, Richard Wirth^5^, Martin J. Whitehouse^6^, Daniel J. Dunkley^7^, Simon A. Wilde^8^, Dirk Schaumlöffel^9^, Julien Malherbe^9^ and Katie L. Moore^2,10^

^*^corresponding author

^1^Department of Earth and Environmental Sciences, University of Manchester, UK ([Ian.Lyon@manchester.ac.uk](mailto:Ian.Lyon@manchester.ac.uk))

^2^Photon Science Institute, University of Manchester, UK

^3^Institute of Geological Sciences, Polish Academy of Sciences, PL-00818 Warsaw, Poland ([mkusiak@twarda.pan.pl](mailto:mkusiak@twarda.pan.pl))

^4^GeoForschungsZentrum, Section 3.6 Chemistry and Physics of Earth Materials, D-14473 Potsdam, Germany ([mkusiak@gfz-potsdam.de)](mailto:mkusiak@gfz-potsdam.de))

^5^GeoForschungsZentrum, Section 3.5 Interface Geochemistry D-14473 Potsdam, Germany ([wirth@gfz-potsdam.de](mailto:wirth@gfz-potsdam.de))

^6^Swedish Museum of Natural History, SE-104 05 Stockholm, Sweden ([Martin.Whitehouse@nrm.se](mailto:Martin.Whitehouse@nrm.se))

^7^Faculty of Earth Sciences, University of Silesia in Katowice, PL-41205 Sosnowiec, Poland (daniel.dunkley@us.edu.pl)

^8^School of Earth and Planetary Sciences, Curtin University, PO BOX U1987, WA 6845, Perth, Australia ([s.wilde@curtin.edu.au](mailto:s.wilde@curtin.edu.au))

^9^CNRS/Université de Pau et des Pays de l’Adour, E2S UPPA, IPREM, UMR 5254, 64000 Pau, France ([dirk.schaumloeffel@univ-pau.fr](mailto:dirk.schaumloeffel@univ-pau.fr))

^10^Department of Materials, University of Manchester, UK ([Katie.Moore@manchester.ac.uk](mailto:Katie.Moore@manchester.ac.uk))

**Table 1. ^207^Pb/^206^Pb ratios acquired from individual discrete nanospheres and from zircon host around the nanospheres. A ratio of ‘zero’ results from zero counts for that species in the region of interest defined in the analysis area. ‘n.a.’ indicates ‘not available’, detectors were set to acquire ions for ^207^Pb and ^206^Pb only and not ^204^Pb or ^208^Pb.**

**Sample 975 grain 66**

**Nanospheres**

| ^207^Pb^/206^Pb | ^208^Pb^/206^Pb | ^204^Pb^/206^Pb |
| --- | --- | --- |
| 0.542 ± 0.042 | 0.030 ± 0.015 | 0.008 ± 0.008 |
| 0.533 ± 0.072 | 0.052 ± 0.037 | 0 |
| 0.397 ± 0.059 | 0.171 ± 0.066 | 0 |
| 0.403 ± 0.054 | 0.060 ± 0.035 | 0 |
| 0.448 ± 0.048 | 0.054 ± 0.027 | 0.014 ± 0.014 |
| 0.547 ± 0.045 | 0.060 ± 0.024 | 0.010 ± 0.010 |
| 0.467 ± 0.040 | 0.086 ± 0.029 | 0 |
| 0.581 ± 0.061 | n.a. | n.a. |
| 0.475 ± 0.068 | n.a. | n.a. |
| 0.533 ± 0.063 | n.a. | n.a. |
| 0.552 ± 0.065 | n.a. | n.a. |
| 0.564 ± 0.070 | n.a. | n.a. |
| 0.700 ± 0.100 | n.a. | n.a. |
| 0.590 ± 0.055 | n.a. | n.a. |
| 0.493 ± 0.042 | n.a. | n.a. |
| 0.453 ± 0.046 | n.a. | n.a. |
| 0.533 ± 0.047 | n.a. | n.a. |
| 0.422 ± 0.044 | n.a. | n.a. |
| 0.533 ± 0.073 | n.a. | n.a. |
| 0.603 ± 0.105 | n.a. | n.a. |
| 0.401 ± 0.056 | n.a. | n.a. |
| 0.460 ± 0.070 | n.a. | n.a. |
| 0.643 ± 0.196 | n.a. | n.a. |
| 0.446 ± 0.025 | n.a. | n.a. |
| 0.597 ± 0.041 | n.a. | n.a. |
| 0.573 ± 0.062 | n.a. | n.a. |
| 0.493 ± 0.040 | n.a. | n.a. |
| 0.521 ± 0.040 | n.a. | n.a. |
| 0.600 ± 0.069 | n.a. | n.a. |
| 0.538 ± 0.046 | n.a. | n.a. |
| 0.531 ± 0.049 | n.a. | n.a. |
| 0.550 ± 0.044 | n.a. | n.a. |
| 0.539 ± 0.047 | n.a. | n.a. |
| 0.540 ± 0.038 | n.a. | n.a. |
| 0.526 ± 0.051 | n.a. | n.a. |
| 0.468 ± 0.040 | 0.046 ± 0.032 | 0 |
| 0.685 ± 0.072 | 0.045 ± 0.045 | 0 |
| 0.685 ± 0.072 | 0.045 ± 0.045 | 0 |
| 0.627 ± 0.053 | 0.042 ± 0.017 | 0 |
| 0.575 ± 0.052 | 0.061 ± 0.022 | 0 |
| 0.398 ± 0.043 | 0.0410 ± 0.018 | 0.025 ± 0.014 |
| 0.462 ± 0.070 | 0.018 ± 0.018 | 0 |
| 0.501 ± 0.075 | 0.038 ± 0.027 | 0 |
| 0.643 ± 0.082 | 0.016 ± 0.016 | 0 |
| 0.423 ± 0.063 | 0.049 ± 0.029 | 0 |
| 0.283 ± 0.018 | 0.044 ± 0.010 | 0.007 ± 0.004 |
| 0.246 ± 0.022 | 0.020 ± 0.009 | 0.004 ± 0.004 |
| 0.269 ± 0.032 | 0.044 ± 0.018 | 0.022 ± 0.013 |
| 0.611 ± 0.027 | 0.015 ± 0.007 | 0 |
| 0.657 ± 0.028 | 0.033 ± 0.011 | 0 |
|  |  |  |
| **Weighted mean = 0.528** |  |  |
| **σ = 0.079** |  |  |
| **St. err = 0.012** |  |  |
|  |  |  |

**Sample 975 grain 66**

**Zircon host areas around nanospheres**

| **^207^Pb^/206^Pb** | **^208^Pb^/206^Pb** | **^204^Pb^/206^Pb** |
| --- | --- | --- |
| 0.151 ± 0.051 | 0 | 0 |
| 0.219 ± 0.030 | 0.216 ± 0.055 | 0 |
| 0.148 ± 0.048 | 0.053 ± 0.053 | 0.053 ± 0.053 |
| 0.156 ± 0.012 | n.a. | n.a. |
| 0.170 ± 0.034 | n.a. | n.a. |
| 0.151 ± 0.033 | n.a. | n.a. |
| 0.130 ± 0.015 | n.a. | n.a. |
| 0.171 ± 0.020 | n.a. | n.a. |
| 0.177 ± 0.009 | n.a. | n.a. |
| 0.208 ± 0.013 | n.a. | n.a. |
|  |  |  |
| **Weighted mean = 0.170** |  |  |
| **σ = 0.026** |  |  |
| **St. err = 0.008** |  |  |

**Sample 975 Grain 07**

**Nanospheres**

| ^207^Pb^/206^Pb | ^208^Pb^/206^Pb | ^204^Pb^/206^Pb |
| --- | --- | --- |
| 0.567 ± 0.049 | 0.049 ±0.034 | 0 |
| 0.630 ± 0.206 | 0 | 0.251 ±0.251 |
| 0.544 ± 0.116 | 0.158 ± 0.158 | 0 |
| 0.527 ± 0.121 | 0 | 0 |
| 0.548 ± 0.112 | 0.527 ± 0.313 | 0 |
| 0.353 ± 0.106 | 0 | 0 |
| 0.387 ± 0.097 | 0.374 ± 0.270 | 0 |
| 0.298 ± 0.102 | 0 | 0 |
| 0.474 ± 0.109 | 0.320 ± 0.230 | 0 |
| 0.500 ± 0.076 | 0 | 0 |
| 0.488 ± 0.069 | 0.054 ± 0.054 | 0 |
| 0.311 ± 0.061 | 0.150 ± 0.106 | 0 |
| 0.694 ± 0.054 | 0 | 0 |
| 0.555 ± 0.041 | 0 | 0 |
| 0.593 ± 0.056 | 0 | 0 |
| 0.537 ± 0.054 | 0 | 0 |
| 0.556 ± 0.065 | 0 | 0.043 ± 0.043 |
| 0.408 ± 0.053 | 0 | 0 |
| 0.582 ± 0.057 | 0 | 0 |
| 0.321 ± 0.132 | 0 | 0 |
| 0.515 ± 0.208 | 0 | 0 |
| 0.576 ± 0.107 | 0 | 0 |
| 0.511 ± 0.098 | 0 | 0 |
| 0.532 ± 0.115 | 0 | 0 |
| 0.611 ± 0.028 | 0.018 ± 0.006 | 0.002 ±0.002 |
| 0.593 ± 0.023 | 0.019 ± 0.005 | 0.001 ± 0.001 |
| 0.504 ± 0.036 | 0.009 ± 0.006 | 0.004 ± 0.004 |
| 0.522 ± 0.031 | 0.028 ± 0.009 | 0 |
| 0.525 ± 0.034 | 0.030 ± 0.010 | 0.007 ± 0.005 |
| 0.592 ± 0.036 | 0.010 ± 0.006 | 0 |
| 0.540 ± 0.037 | 0.016 ± 0008 | 0.004 ± 0.004 |
| 0.456 ± 0.038 | 0.011 ± 0.008 | 0.006 ± 0.006 |
| 0.510 ± 0.039 | 0.005 ± 0.005 | 0 |
| 0.527 ± 0.037 | 0.022 ± 0.010 | 0 |
| 0.440 ± 0.040 | 0.020 ± 0.010 | 0 |
| 0.567 ± 0.049 | 0.027 ± 0.014 | 0.013 ±0.009 |
| 0.552 ± 0.042 | 0.015 ± 0.009 | 0.005 ± 0.005 |
| 0.646 ± 0.052 | 0.030 ± 0.015 | 0.013 ± 0.009 |
|  |  |  |
| **Weighted mean = 0.495** |  |  |
| **σ = 0.112** |  |  |
| **St. err = 0.016** |  |  |

**Sample 975 grain 07**

**Zircon host around nanospheres**

| **^207^Pb^/206^Pb** | **^208^Pb^/206^Pb** | **^204^Pb^/206^Pb** |
| --- | --- | --- |
| 0.204 ± 0.033 | 0 | 0 |
| 0.200 ± 0.054 | 0 | 0 |
| 0.193 ± 0.009 | 0.027 ± 0.005 | 0.031 ± 0.006 |
| 0.211 ± 0.013 | 0.027 ± 0.007 | 0.007 ± 0.004 |
| 0.185 ± 0.008 | 0.027 ± 0.004 | 0.023 ± 0.004 |
| **Weighted mean = 0.195** |  |  |
| **σ = 0.010** |  |  |
| **St. err = 0.004** |  |  |


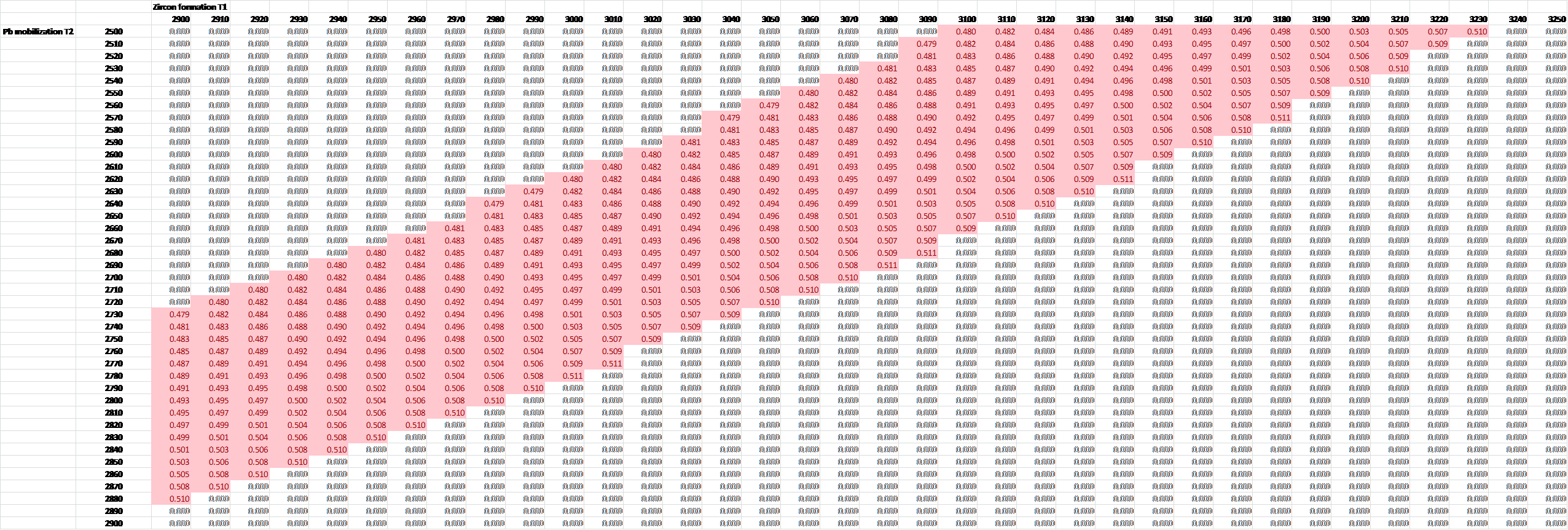


Figure 1a. Model ages within the pink highlighted band can give the ^207^Pb/^206^Pb ratios measured in nanospheres and zircon host in grains 66 and 07.


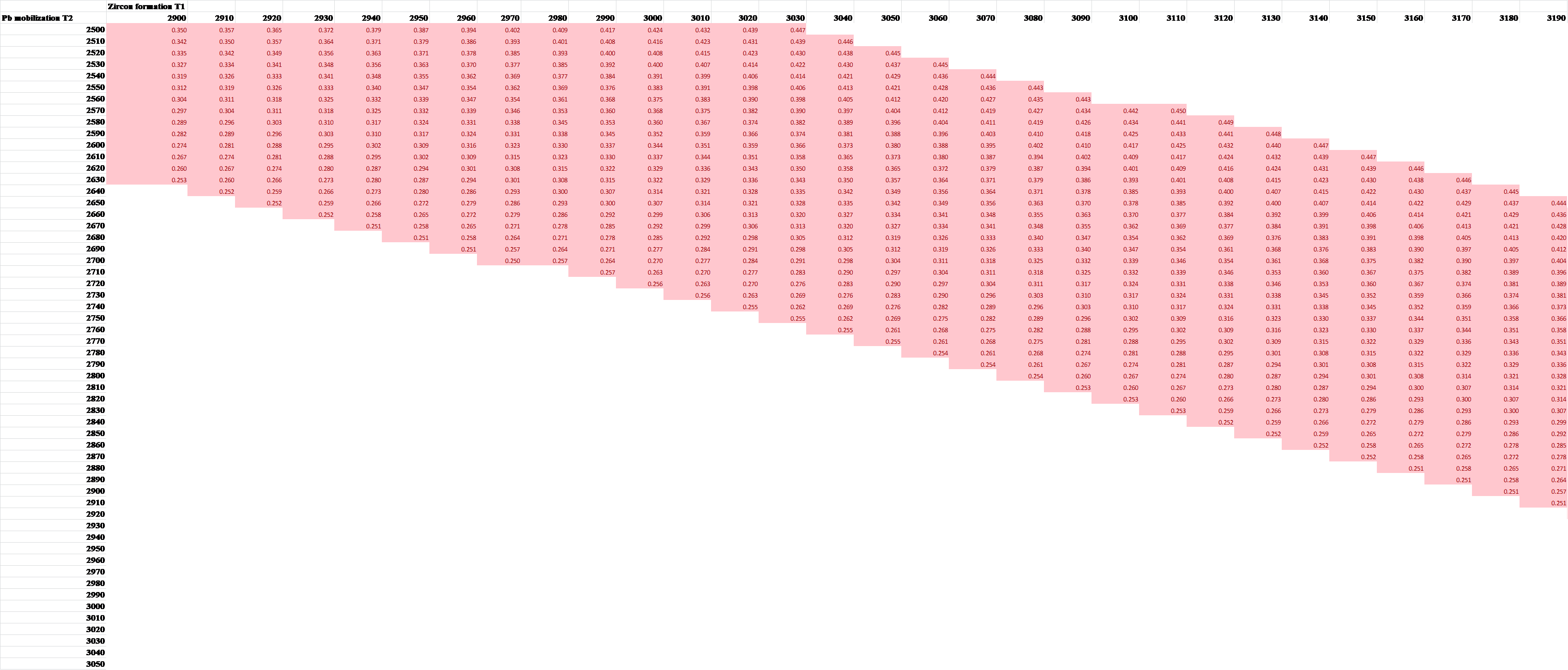


Figure 1b. Model T_1_ and T_2_ ages which satisfy the measured abundance ratio of Pb in nanospheres to Pb in the host zircon.


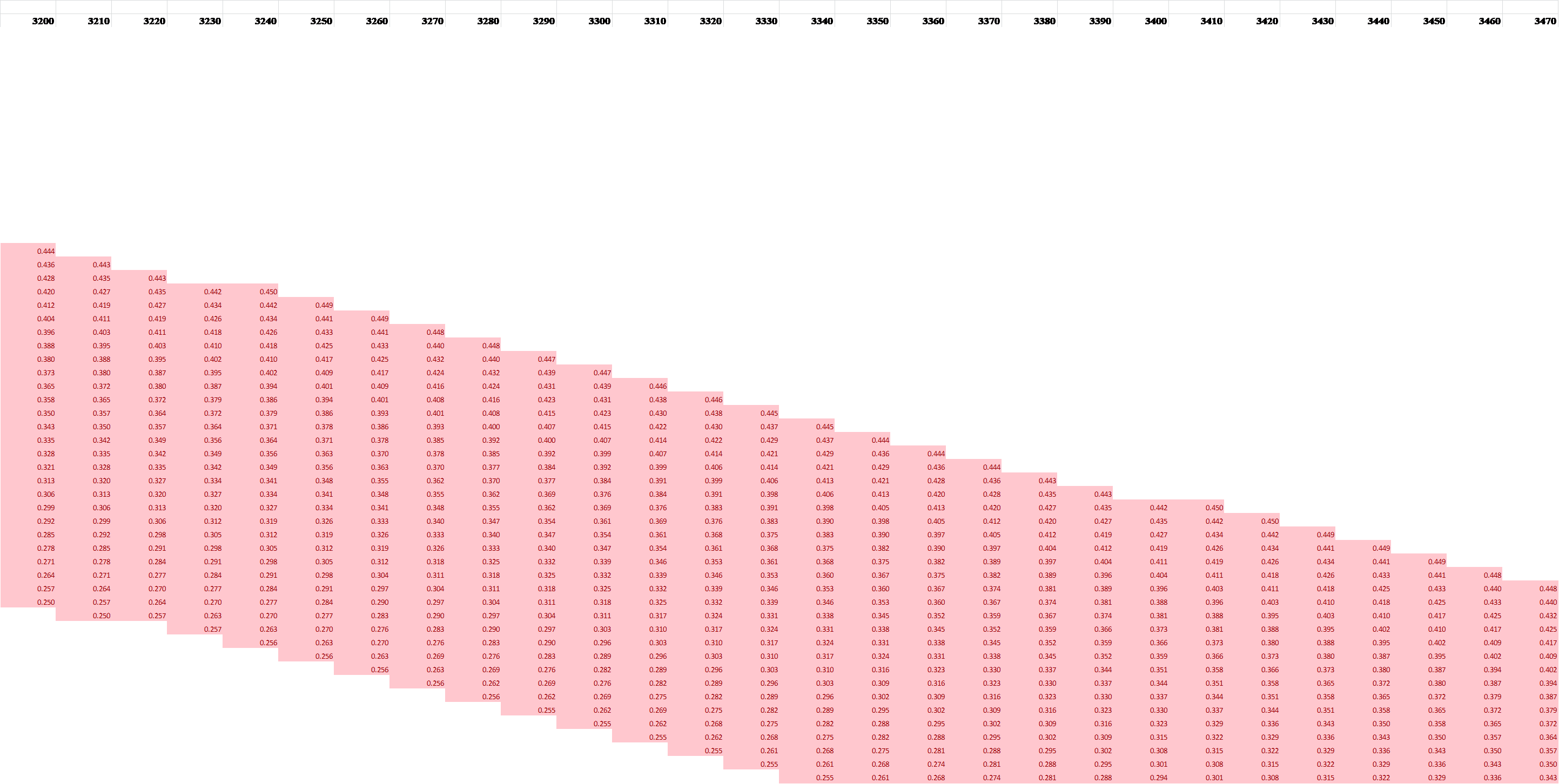


Fig. 1b-continued. Range of model T1 and T2 which give the experimentally measured ratio of Pb in nanospheres to Pb in the zircon host.

Figure 1c. Range of model T_1_ and T_2_ (pink area) which satisfy the measured ^207^Pb/^206^Pb ratios in nanospheres and host zircon and also satisfy the measured Pb abundance ratio between nanospheres and the host zircon.





Figure 2. ^40^Ca^+^ map acquired at the same time and covering the same area as the images in figure 2 of the main paper.
